# Providers’ Perspectives on Telemental Health Usage After the COVID-19 Pandemic: Retrospective Analysis

**DOI:** 10.2196/39634

**Published:** 2022-11-11

**Authors:** Hattie Wilczewski, Samantha R Paige, Triton Ong, Hiral Soni, Janelle F Barrera, Brandon M Welch, Brian E Bunnell

**Affiliations:** 1 Doxy.me Research Doxy.me Inc Rochester, NY United States; 2 Department of Psychiatry and Behavioral Neurosciences University of South Florida Tampa, FL United States; 3 Biomedical Informatics Center Medical University of South Carolina Charleston, SC United States

**Keywords:** telemedicine, telehealth, COVID-19, telemental health, mental health, pandemic, perception, use, usefulness, usage, workflow

## Abstract

**Background:**

Mental health care pivoted to telemedicine during the COVID-19 pandemic, and there is uncertainty around the sustainability of this rapid shift.

**Objective:**

This study examined how intentions to continue using telemedicine after the COVID-19 pandemic are influenced by provider perceptions of usefulness, ease of use, and professional social influence, facilitating organizational conditions.

**Methods:**

We conducted a web-based, cross-sectional survey of 369 telemental health providers between February and March 2021. A hierarchical linear regression analysis was conducted to predict intentions to continue using telemedicine after the COVID-19 pandemic.

**Results:**

Most providers began using telemedicine in March 2020 or later (257/369, 69.6%) and attended to ≥50% of their clients via telemedicine (299/369, 81.0%). Intention to continue using telemedicine after the COVID-19 pandemic was predicted by the telemedicine caseload (β=.10; *P*=.005), perceived usefulness in general (β=.10; *P*=.008), ease of use (β=.08; *P*=.04), social influence (β=.68; *P*<.001), and facilitating conditions (β=.08; *P*=.047).

**Conclusions:**

Exploration of the predictors of telemedicine usage beyond the COVID-19 pandemic aids in surveillance of telemedicine usage, integration with future clinic workflows, and the shaping of public policy. It is important to consider telemedicine services as not only a response to a crisis but also an effective and useful solution for everyday life. Our results suggest widespread, sustainable telemedicine adoption.

## Introduction

The COVID-19 pandemic significantly impacted mental health (MH) care systems, leading providers to transition rapidly to remote care delivery [1]. In early 2020, in-person mental health care decreased by 50%-70% [2,3] as telemedicine usage increased as much as 6500% [3,4]. This required MH providers to adjust to new technology and loss of in-person care [5] but proved satisfactory for patients owing to decreased wait time, travel time, and absenteeism from work [6,7]. Increased access to patients in rural regions and those with practical barriers to access mental health care may encourage providers’ long-term integration of telemedicine into their practice [8]. Telemental health (TMH) emerged as a response to a crisis and has proven to be an effective, useful, and sustainable form of health care delivery [9]. However, widespread adoption of telemedicine was largely due to emergency regulations providing coverage during the pandemic. There is some concern of a telehealth cliff, or sudden reversal of TMH usage once emergency regulations are lifted [10,11]. Toward this end, we aim to examine how TMH providers perceive the general and pandemic-specific usefulness of TMH and how this relates to their intention to use it in the future.

Behavioral intention is one of the strongest predictors of sustaining or changing a behavior [12]. Studies from both early 2019 [13] and August 2020 [14] found that TMH providers intended to use telemedicine more often in the future. In a study conducted several months before the COVID-19 pandemic, TMH providers voiced concerns about security and technological difficulties but indicated that the benefits to care and workflow strengthened their intentions to use it in the future [13]. Providers have reported other benefits such as improved work-life balance, more flexibility in scheduling, and being able to deliver innovative care [13,15]. During the pandemic, providers who served rural areas and were reimbursed through self-pay methods reported the greatest intentions to use telemedicine in the future, which may be attributed to greater comfort with using the technology as a result of more frequent use [14]. Further, one study found that psychologists anticipated a 5-fold increase in telemedicine usage from prepandemic rates [16]. There is a need to continue to understand MH providers’ experiences and intentions as the pandemic evolves.

There are many barriers to integrating telemedicine services into regular care, including technology acceptance [17-19]. The Technology Acceptance Model [20] (TAM) and Unified Theory of Acceptance and Use of Technology [21] (UTAUT) are applied commonly to understand MH providers’ use of telemedicine, but not all constructs in the TAM and UTAUT have equal predictive weight. Perceptions about the usefulness of telemedicine are grounded in how the technology can improve care [20,21]; that is, usefulness can be thought of in terms of the benefits the technology brings to providers (eg, reducing no-show rates, reducing costs or overhead, and improving work-life balance) [20,22]. Furthermore, perceptions about ease-of-use are generally based on design features and functionality to facilitate tasks such as coordinating care plans or effectively communicating with patients, patient families, and other health care providers [19]. Effort expectancy has been used synonymously with ease of use [23,24]. The effect of these perceptions on intentions to use telemedicine in the future have yet to be explored among mental health providers after COVID-19 uptake.

Previous studies have found that perceived usefulness was the strongest predictor of telemedicine adoption among MH providers along with perceived ease of use, social influence, and attitude [24,25]. Perceived usefulness from the telemedicine provider perspective refers to quality of care, diagnosis, and monitoring [24,26,27]. Social influence is the degree to which an individual’s decision to use a technology is influenced by others’ perceptions of the technology [24]. Social influence from other providers may shape intention to integrate a technology into one’s own practice, especially when driven by the competitive desire to deliver cutting-edge, innovative care [28]. Thus, the effect of social influence from one’s peers on TMH adoption warrants continued investigation [29].

MH providers report that they are still providing high-quality care and effectively communicating with their patients despite the abrupt transition to telemedicine during the COVID-19 pandemic [30]. However, the effect of facilitating conditions (ie, the amount of support, resources, and training available from one’s organization to effectively practice their specialty via telemedicine) on this relation has received limited attention [24,31]. To optimize resources dedicated to increasing high-quality telemedicine use in MH care, there is a need to identify the “active ingredients” that predict providers’ intentions to use telemedicine in the future.

Providers are the gatekeepers of telemedicine [32,33], warranting continued investigation into provider preferences of usage beyond the COVID-19 pandemic. Knowing the characteristics of MH providers who intend to use telemedicine after the COVID-19 pandemic is important for surveillance of telemedicine utilization and for efforts directed toward strengthening hesitant providers’ intentions. Therefore, the purpose of this study is to further investigate determinants of TMH providers’ intentions to continue using telemedicine beyond the COVID-19 pandemic.

## Methods

### Recruitment

Providers were invited to participate in a cross-sectional, web-based survey between February and March 2021, the primary results of which are published elsewhere [34]. Eligible providers included English-speaking, adult (≥18 years of age), mental health providers in the United States. Providers were registered with Doxy.me Inc [35], a commercial telemedicine company that offers secure telecommunications technology for providers to use in their own practice. In total, 495 providers agreed to participate in the study, 369 of whom had complete data for the purposes of this analysis (74.5% completion rate) [36]. The demographic characteristics of providers in the sample were consistent with those of providers in the mental health industry in the United States [13,14,30,34,37,38].

The survey was distributed to participants through email and administered via Qualtrics (Qualtrics Inc). The survey began with an electronic consent form detailing that deidentified data would be used for publication and that 1 free month of a Doxy.me professional membership account would be offered in compensation for time spent completing the survey.

### Ethical Considerations

The study was reviewed and deemed exempt by the institutional review board of the University of South Florida (IRB#002053).

### Survey and Measures

#### Overview

The research team iteratively refined and developed the survey exploring several aspects related to TMH practice before, during, and after the COVID-19 pandemic. Participants were asked to provide personal (eg, age, gender, race, and ethnicity) and clinical (eg, practice type, specialty, reimbursement, and treatment paradigm) demographics. They were then asked about their perceptions toward telemedicine relating to perceived usefulness, perceived ease of use, social influence, facilitating conditions, and intention to use telemedicine after the pandemic. Multimedia Appendix 1 provides the survey questions and response frequencies.

Several demographic variables were recoded during analysis. Professional title and practice type variables were recoded to incorporate responses in the “other” category. Telemedicine caseload was dichotomized to “<50%” (including choices of <25% and 25%-49%) versus “≥50%” (including choices of 50%-75% and >75%). Further, the onset of telemedicine usage was dichotomized to “before March 2020” (combining “December 2019 or earlier” and “January or February 2020”) versus “March 2020 or later.”

#### Perceived Usefulness

Perceived usefulness was assessed using 2 scales. The first scale included the average of 3 items measuring provider perspectives about the *general benefits of telemedicine* (questions 27-29 in Multimedia Appendix 1). The second scale included the average of 3 items measuring provider perspectives about the *benefits of telemedicine specifically in relation to COVID-19* (questions 24-26 in Multimedia Appendix 1). Responses to all items were anchored on a 5-point Likert scale (1=*Not at all* to 5=*Extremely*). Both scales had adequate internal consistency (Cronbach =.70-.81).

#### Perceived Ease of Use

Perceived ease of use was assessed using the System Usability Scale (SUS) [39], which includes 10 items that are alternatingly worded positively (eg, “I think telemedicine is easy to use in my practice”) and negatively (eg, “I find telemedicine unnecessarily complex”). Responses were anchored on a 5-point Likert Scale (1=*Strongly Disagree* to 5=*Strongly Agree*), and the SUS score was determined by rescaling the responses, resulting in a single score between 0 and 100. Internal consistency was adequate for the SUS scale (Cronbach =.77). The SUS items are detailed in Multimedia Appendix 1 (questions 14-23).

#### Social Influence

Social influence by other mental health providers was measured using 1 item (ie, “After the COVID-19 pandemic is resolved, I expect telemedicine to continue to be used by others in my profession”), which was anchored on a 5-point Likert scale from 1=*Much less* to 5=*Much more* (question 34 in Multimedia Appendix 1).

#### Facilitating Conditions

Facilitating conditions were measured by calculating the mean of 4 items asking about the extent to which providers felt comfortable, supported, trained, and adequately resourced by their practice relative to providing telemedicine services (questions 30-33 in Multimedia Appendix 1). Responses were anchored on a 5-point Likert scale from 1=*Strongly disagree* to 5=*Strongly agree*. Internal consistency was adequate for the facilitating conditions scale (Cronbach =.78).

#### Intentions to Continue Using Telemedicine After the COVID-19 Pandemic

Intentions to continue using telemedicine after the COVID-19 pandemic were measured using 1 item (ie, “After the COVID-19 pandemic is resolved, I expect to use telemedicine in my practice…”). Responses were anchored on a 5-point Likert scale from 1=*Much less* to 5=*Much more* (question 35 in Multimedia Appendix 1).

### Statistical Analysis

SPSS (version 28; IBM Corp) was used for all analyses. Significance was determined with a 2-tailed α of <.05. We conducted independent samples *t* tests to examine differences in intentions to use telemedicine post the COVID-19 pandemic, based on telemedicine caseload (either <50% or ≥50% of patients served remotely), the onset of telemedicine usage in relation to the COVID-19 pandemic (either before March 2020 or during and after March 2020), gender (male or female), and ethnicity (Hispanic or non-Hispanic).

We conducted a 1-way ANOVA to determine differences in intentions to use telemedicine post the COVID-19 pandemic by race. Next, we conducted a correlation analysis to examine the relation between age and intentions to continue using telemedicine after the COVID-19 pandemic. Lastly, we conducted a 5-step hierarchical linear regression analysis to examine the predictive power of provider characteristics (ie, telemedicine caseload, onset of telemedicine usage in relation to the COVID-19 pandemic, and age) and perceptions toward telemedicine (ie, perceived usefulness, perceived ease-of-use, social influence, and facilitating conditions) with respect to intentions to continue using telemedicine after the COVID-19 pandemic. Each step in the regression analysis added a block of variables, determining incremental prediction above and beyond variables in the previous steps. Telemedicine caseload (0=<50%; 1=≥50%), onset of telemedicine usage (0=before March 2020; 1=March 2020 or later), and age were included in step 1 of the model as covariates because they were significantly associated with the intention to use telemedicine after the COVID-19 pandemic. The next step included perceived usefulness variables (eg, general perceived usefulness and perceived usefulness related to the COVID-19 pandemic), with step 3 adding ease of use, step 4 adding social influence, and step 5 adding facilitating conditions. ANOVAs were conducted to determine model comparisons.

## Results

### Sample Characteristics

The mean age of providers was 52 (SD 13.0) years. Most providers identified as female (300/369, 81.3%), White (298/369, 80.8%), and non-Hispanic (339/369, 91.9%). Professionally, most providers were mental health counselors (179/369, 48.5%), psychologists (108/369, 29.3%), or social workers (54/369, 14.6%) working in individual practice settings (279/369, 75.6%) and primarily treating adult patients (18-64 years old; 308/369, 83.5%). More details regarding provider and practice characteristics are reported elsewhere [34].

### Characteristics of Telemedicine Practice and Measures

As shown in Table 1, a total of 257 of 369 (69.6%) providers began using telemedicine in March 2020 or later, and 299 (81.0%) reported that more than ≥50% of their caseload was attended to via telemedicine. With respect to intentions to continue using telemedicine after the COVID-19 pandemic, 173 (46.9%) intended to use it more, 66 (17.9%) intended to use it about the same, and 130 (35.2%) intended to use it less. Most providers (193/369, 52.3%) expected telemedicine to be used more by others in their profession, while fewer providers (115/369, 31.2%) expected their colleagues to use it about the same (61/369, 16.5%) or less.

Providers who attended to ≥50% of clients via telemedicine reported stronger intentions to continue using telemedicine after the COVID-19 pandemic (mean 3.4, SD 1.4) than those who attended to <50% of clients via telemedicine (mean 2.6, SD 1.3; *t*_367_=–4.07; *P*<.001). Providers who began to use telemedicine before March 2020 also reported stronger intentions to continue using telemedicine after the COVID-19 pandemic (mean 3.5, SD 1.3) than those who began using it in March 2020 or later (mean 3.1, SD 1.4; *t*_367_=2.46; *P*=.01). Provider age was negatively correlated with intentions to continue using telemedicine after the COVID-19 pandemic (*r*_367_=–0.11; *P*=.03). There were no significant differences in intentions to use telemedicine after the COVID-19 pandemic by gender (*t*_365_=–0.84; *P*=.40), race (*F*_6,360_=0.431; *P*=.86), or ethnicity (*t*_367_=0.26; *P*=.80). In subsequent analyses, telemedicine caseload, duration of usage, and age were included as covariates to be controlled for in the hierarchical regression analysis. Multimedia Appendix 2 shows the correlation table of covariates and measures.

**Table 1:** Characteristics of telemedicine practices and measures (N=369).

Demographics		Values
**Onset of telemedicine usage, n (%)**		
	Before March 2020	112 (30.4)
	March 2020 or later	257 (69.6)
**Telemedicine caseload, n (%)**		
	<50%	70 (19.0)
	≥50%	299 (81.0)
**Measures, mean (SD)**		
	Perceived usefulness (general)	3.07 (1.11)
	Perceived usefulness (COVID-19)	4.53 (0.67)
	Perceived ease of use	75.26 (14.87)
	Social influence	3.47 (1.36)
	Facilitating conditions	4.11 (0.76)
	Intentions to continue using telemedicine after the COVID-19 pandemic	3.24 (1.42)

### Predicting Intentions to Continue Using Telemedicine After the COVID-19 Pandemic

Table 2 displays the results for each step of the hierarchical linear regression analysis and Table 3 shows the results for predictors in each step of the model. Step 1 of the model was significant and accounted for 7% of the variance in intentions to use telemedicine after the COVID-19 pandemic (*F*_3,365_=9.12; *P*<.001; *R*^2^=0.07). Telemedicine caseload (β=.21; *P*<.001) and onset of telemedicine usage (β=–.14; *P*=.007) significantly predicted intentions to continue using telemedicine after the COVID-19 pandemic. Age was not a significant predictor (*P*=.09).

Step 2 included the addition of perceived usefulness to the model. Controlling for the effects of telemedicine caseload, onset of usage, and age, the regression was significant and accounted for an additional 13% of the variance in intentions to continue using telemedicine after the COVID-19 pandemic (*F*_5,363_=17.71; *P*<.001; *R*^2^=0.20; Δ*R*^2^=0.13). Onset of telemedicine usage (β=–.12; *P*=.009), perceived usefulness in general (β=.31; *P*<.001) and in relation to the COVID-19 pandemic (β=.11; *P*=.04) significantly predicted intentions to use telemedicine after the COVID-19 pandemic. Age and telemedicine caseload were not significant predictors (*P*>.05 for all).

Step 3 included the addition of perceived ease of use to the model. Controlling for the effects of covariates and perceived usefulness, the regression was significant and accounted for an additional 3% of the variance in intentions to continue using telemedicine after the COVID-19 pandemic (*F*_6,362_=17.56; *P*<.001; *R*^2^=0.23; Δ*R*^2^=0.03). Onset of telemedicine usage (β=–.11; *P*=.02), perceived usefulness in general (β=.27; *P*<.001), and perceived ease of use (β=.18; *P*<.001) significantly predicted intentions to continue using telemedicine after the COVID-19 pandemic. Age, telemedicine caseload, and perceived usefulness in relation to the COVID-19 pandemic were not significant predictors (*P*>.05 for all).

Step 4 included the addition of social influence to the model. Controlling for the effects of covariates and perceived usefulness and ease of use, the regression was significant and accounted for an additional 41% of the variance in intentions to continue using telemedicine after the COVID-19 pandemic (*F*_7,361_=91.03; *P*<.001; *R*^2^=0.64; Δ*R*^2^=0.41). Onset of telemedicine usage (β=–.06; *P*=.02), telemedicine caseload (β=–.10; *P*=.005), perceived usefulness in general (β=.11; *P*=.004), ease of use (β=.11; *P*=.001), and social influence (β=.69; *P*<.001) significantly predicted intentions to use telemedicine after the COVID-19 pandemic. Age and perceived usefulness in relation to the COVID-19 pandemic were not significant predictors (*P*>.05 for all).

Step 5 included the addition of facilitating conditions to the model. Controlling for the effects of covariates, perceived usefulness and ease of use, and social influence, the regression was significant and accounted for an additional 0.4%, for a total of 64% of the variance in intentions to continue using telemedicine after the COVID-19 pandemic (*F*_8,360_=80.80; *P*<.001; *R*^2^=0.64; Δ*R*^2^=0.004). Telemedicine caseload (β=.10; *P*=.005), perceived usefulness in general (β=.10; *P*=.008), ease of use (β=.08; *P*=.04), social influence (β=.68; *P*<.001), and facilitating conditions (β=.08; *P*=.047) significantly predicted intentions to use telemedicine after the COVID-19 pandemic. Age, onset of telemedicine usage, and perceived usefulness in relation to the COVID-19 pandemic were not significant predictors (*P*>.05 for all).

**Table 2:** Model comparisons for each step of the hierarchical regression analysis.

Step^a^	*F* test (*df*)	*R* ^2^	Adjusted *R*^2^	Δ*R*^2^
1	9.12 (3,365)	0.07	0.06	0.07
2	17.71 (5,363)	0.20	0.19	0.13
3	17.56 (6,362)	0.23	0.21	0.03
4	91.03 (7,361)	0.64	0.63	0.41
5	80.80 (8,360)	0.64	0.63	0.004

^a^*P*<.001 for all steps and Δ*R^2^*.

**Table 3:** Predictors of intentions to use telemedicine after the COVID-19 pandemic.

Predictor			B (SE)		95% CI		β		*t* test (*df*)		*P* value
**Step 1**											
	Constant	2.67 (0.48)		1.73 to 3.61		Reference		5.61 (365)		<.001	
	Telemedicine caseload	0.74 (0.18)		0.38 to 1.10		.21		4.04 (365)		<.001	
	Onset of telemedicine usage	–0.42 (0.16)		–0.73 to –0.12		–.14		–2.71 (365)		.007	
	Age	–0.01 (0.01)		–0.02 to –0.002		–.09		–1.68 (365)		.09	
**Step 2**											
	Constant	0.97 (0.61)		–0.22 to 2.17		Reference		1.60 (363)		.11	
	Telemedicine caseload	0.35 (0.18)		0.001 to 0.71		.10		1.96 (363)		.05	
	Onset of telemedicine usage	–0.38 (0.15)		–0.67 to –0.09		–.12		–2.61 (363)		.009	
	Age	–0.01 (0.01)		–0.02 to 0.002		–.07		–1.53 (363)		.13	
	Usefulness (general)	0.40 (0.07)		0.27 to 0.53		.31		5.93 (363)		<.001	
	Usefulness (COVID-19)	0.24 (0.11)		0.02 to 0.46		.11		2.11 (363)		.04	
**Step 3**											
	Constant	0.08 (0.64)		–1.19 to 1.35		Reference		0.12 (362)		.90	
	Telemedicine caseload	0.35 (0.18)		–0.002 to 0.70		.10		1.96 (362)		.05	
	Onset of telemedicine usage	–0.33 (0.14)		–0.61 to –0.05		–.11		–2.30 (362)		.02	
	Age	–0.01 (0.01)		–0.02 to 0.002		–.07		–1.51 (362)		.13	
	Usefulness (general)	0.34 (0.07)		–0.21 to 0.48		.27		5.10 (362)		<.001	
	Usefulness (COVID-19)	0.17 (0.11)		–0.05 to 0.40		.08		1.56 (362)		.12	
	Ease of use	0.02 (0.005)		0.01 to 0.03		.18		3.70 (362)		<.001	
**Step 4**											
	Constant	–1.15 (0.44)		–2.02 to –0.27		Reference		–2.58 (361)		.01	
	Telemedicine caseload	0.34 (0.12)		0.11 to 0.58		.10		2.84 (361)		.005	
	Onset of telemedicine usage	–0.20 (0.10)		–0.39 to –0.004		–.06		–2.01 (361)		.045	
	Age	–0.001 (0.004)		–0.01 to 0.01		–.01		–0.27 (361)		.79	
	Usefulness (general)	0.14 (0.05)		0.04 to 0.23		.11		2.90 (361)		.004	
	Usefulness (COVID-19)	0.05 (0.08)		–0.10 to 0.20		.03		0.69 (361)		.49	
	Ease of use	0.01 (0.003)		0.004 to 0.02		.11		3.27 (361)		.001	
	Social influence	0.72 (0.04)		0.65 to 0.79		.69		20.30 (361)		<.001	
**Step 5**											
	Constant	–1.39 (0.46)		–2.29 to –0.48		Reference		–3.02 (360)		.003	
	Telemedicine caseload	0.36 (0.12)		0.12 to 0.59		.10		2.94 (360)		.004	
	Onset of telemedicine usage	–0.18 (0.10)		–0.37 to 0.01		–.06		–1.82 (360)		.07	
	Age	–0.001 (0.004)		–0.01 to 0.01		–.01		–0.26 (360)		.79	
	Usefulness (general)	0.13 (0.05)		0.03 to 0.22		.10		2.65 (360)		.008	
	Usefulness (COVID-19)	0.03 (0.08)		–0.12 to 0.18		.02		0.42 (360)		.68	
	Ease of use	0.01 (0.004)		0.00 to 0.01		.08		2.08 (360)		.04	
	Social influence	0.71 (0.04)		0.64 to 0.78		.68		20.26 (360)		<.001	
	Facilitating conditions	0.14 (0.07)		0.002 to 0.28		.08		1.99 (360)		.047	

## Discussion

The findings of this study show that perceived usefulness in general, perceived ease of use, professional social influence, facilitating conditions, and telemedicine caseload predict MH providers’ intentions to use telemedicine in the future. Age, onset of telemedicine usage, and perceived usefulness in relation to the COVID-19 pandemic were not found to be significant predictors in the final model.

### Principal Findings

Social influence from other MH providers was the strongest predictor of intentions to continue using telemedicine after the COVID-19 pandemic. Pre–COVID-19 research found influence from organizational leadership to be less important for health care providers than perceived usefulness, reporting that a main driver of provider intentions was how useful they believed the technology would be for patients [24,40]. Other studies have examined social influence in general but not from others in one’s profession [23,41]. Social context is an important factor for telemedicine acceptance, and researchers have started to identify a need for a “telehealth culture” to share experiences, opinions, and preferences among providers in the same profession [29,42]. Future research should aim to explore the development and impact of a professional telehealth culture since the onset of the COVID-19 pandemic.

Pre–COVID-19 research showed perceived usefulness to be the most important predictor of provider intentions to continue using telemedicine [24,41,43]. In contrast, our study found social influence from other MH providers to be most influential. This may be due to the somewhat mandatory adoption of telemedicine during the COVID-19 pandemic, compared to pre–COVID-19 studies where telemedicine use may have been more voluntary. The pandemic may have shifted MH providers’ priorities from usefulness to other valuations (eg, social influence). Future research should investigate the role of patient preferences in MH providers’ telemedicine usage.

It may be that providers with larger remote caseloads became more proficient with the use of telemedicine through experience. In previous research, increased experience using telemedicine was found to strengthen providers’ positive attitudes toward remote care after acquiring practice, troubleshooting skills, and workflow integration [29]. Providers with more experience using telemedicine may have a better experience as a result of increased comfort and familiarity with telemedicine interfaces and features, and may experience less frustration owing to technological difficulties and incorporating new technology into their practice [44]. Notably, the onset of telemedicine usage was not a significant predictor in the final model, suggesting that the frequency of telemedicine use mattered more than the duration of use. Future research may focus on the relation between self-efficacy and telemedicine use (ie, workflow integration and technical disruptions) and quality of care (ie, therapeutic alliance and communication).

Perceived ease of use was not among the strongest predictors of intentions to use telemedicine. This finding is consistent with recent examinations of UTAUT in relation to the COVID-19 pandemic [18,24,43]. Providers in this sample reported high ease-of-use ratings, which may have been influenced by widespread adoption, larger telemedicine caseloads, and workflow integrations. Although not a particularly strong predictor, facilitating conditions predicted intentions for continued future use of telemedicine. This finding is consistent with telemedicine acceptance research among providers [41,43].

Notably, 75% of providers in this study practiced in individual practice settings and reported strong support, training, resources, and comfort using telemedicine in their practice. Our results suggest that these facilitating conditions may influence sustainability and practicality of long-term integration of telemedicine in one’s practice. Future research should investigate the effect of different practice types on facilitating conditions.

### Limitations

This study contains several limitations. First, data were sampled from users of one commercial telemedicine company, who were compensated with 1 month of free professional membership. This may not be representative of all telemedicine providers and may have biased sampling toward providers who are more interested in telemedicine. Future research should confirm the generalizability of our findings among TMH providers across platforms and in other contexts and countries, and should focus on negative opinions and experiences as well as positive ones. However, previous studies have reported that TMH users sampled from Doxy.me are representative of overall industry demographics, and other studies have reported similar findings regarding future telemedicine usage [15].

Furthermore, the secondary analysis of data excluded some constructs from the TAM and UTAUT models, and some provider demographics may be overrepresented in the sample (eg, individual practice, mental health counselor, cognitive behavioral therapy treatment paradigm, and primarily treats adults). A stratified sampling procedure would ensure equal representation of practice types and provider specialties. As COVID-19 regulations and norms evolve, future researchers should continue to investigate patterns in MH providers’ telemedicine usage and intentions.

### Conclusions

The purpose of this study was to examine TMH providers’ intentions to continue using telemedicine after the COVID-19 pandemic, which changed the landscape of MH care by necessitating the need for service delivery via telemedicine. Most TMH providers reported intentions to continue using telemedicine in their profession between “about the same” or “more” after the COVID-19 pandemic. We speculate that this points toward widespread, sustained telemedicine adoption in the future. Stronger intention for future use was predicted by social influence, perceived usefulness, telemedicine caseload, perceived ease of use, and facilitating conditions. Social influence from others in one’s profession was the strongest predictor of the continued use of telemedicine. Sustained high rates of telemedicine may lead to the development of a “telehealth culture” in which providers can depend on others in their profession for TMH training, resources, and workflow systems. Rates of current telemedicine usage are expected to remain high even after the COVID-19 pandemic, especially for providers with a large percentage of caseloads seen via telemedicine. Telemedicine appears to be an important part of current and future MH care.

## Appendix

Multimedia Appendix 1 Survey questions and response frequencies.

Multimedia Appendix 2 Correlation table (Pearson r and two-tailed *P* value) of practice characteristics and measures.

## Abbreviations

MH: mental health
SUS: System Usability Scale
TAM: Technology Acceptance Model
TMH: telemental health
UTAUT: Unified Theory of Acceptance and Use of Technology
