# Supplementary material for: Providers’ Perspectives on Telemental Health Usage After the COVID-19 Pandemic: Retrospective Analysis
Source: JMIR Form Res. 2022 Nov 11;6(11):e39634. doi: 10.2196/39634 (PMC9662289; doi:10.2196/39634)
Supplement: Multimedia Appendix 1 [file formative_v6i11e39634_app1.docx]

**Multimedia Appendix 1.**

Survey questions and response frequencies.

1. How do you identify?

- Female (*n* = 300/369, 81.3%)
- Male (*n* = 67/369, 18.2%)
- Other (Please specify): ________ (*n* = 2/369,0.5%)

2. How do you identify racially?

- American Indian/Alaska Native (*n* = 5/369, 1.4%)
- Asian (*n* = 7/369, 1.9%)
- Native Hawaiian or Pacific Islander (*n* = 2/369, 0.5%)
- Black or African American (*n* = 24/369, 6.5%)
- White (*n* =298/369, 80.8%)
- Multiracial (*n* = 17/369, 4.6%)
- Other (Please specify): ________ (*n* = 14/369, 3.8%)
- Missing data (*n* = 2/369, 0.5%)

3. Are you of Hispanic, Latinx, or Spanish origin?

- Yes (*n* = 30/369, 8.1%)
- No (*n* = 339/369, 91.9%)

4. How would you describe the place where you live?

- Metropolitan/City (Urban center) (*n* = 89/369, 24.1%)
- Strong urban influence (*n* = 57/369, 15.4%)
- Moderate urban influence (*n* = 128/369, 34.7%)
- Weak urban influence (*n* = 46/369, 12.5%)
- Rural/Small Town (Remote-no urban influence) (*n* = 49/369, 13.3%)

5. What is your professional title?

- Mental Health Counselor (*n* = 155/369, 42.0%)
- Behavior Analyst (*n* = 0/369, 0.0%)
- Clinical Neuropsychologist (*n* = 3/369, 0.8%)
- Social Worker (*n* = 53/369, 14.4%)
- Marriage and Family Therapist (*n* = 28/369, 7.6%)
- Psychoanalyst (*n* = 1/369, 0.3%)
- Psychologist (*n* = 101/369, 27.4%)
- Psychiatrist (*n* = 0/369, 0.0%)
- Other mental health provider (Please specify): _________ (*n* = 28/369, 7.6%)
- I am not a mental health provider

6. What treatment paradigm do you typically use?

- Behavioral (*n* = 10/369, 2.7%)
- Cognitive-Behavioral (*n* = 208/369, 56.4%)
- Existential/Humanistic (*n* = 38/369, 10.3%)
- Family Systems (*n* = 21/369, 5.7%)
- Interpersonal (*n* = 47/369, 12.7%)
- Psychodynamic/analytic (*n* = 41/369, 11.1%)
- Social Learning (*n* = 4/369, 1.1%)

7. How would you describe your practice?

- Individual practice (*n* = 279/369, 75.6%)
- Network of providers or small clinic (*n* = 70/369, 19.0%)
- Hospital or large clinic (*n* = 12/369, 3.3%)
- School (K-12) (*n* = 3/369, 0.8%)
- College or university (*n* = 2/369, 0.5%)
- Government agency (e.g., Veterans Affairs) (*n* = 3/369, 0.8%)

8. What age group do you primarily treat?

- Children (0-10 yrs old) (*n* = 12/369, 3.3%)
- Adolescents (11-17 yrs old) (*n* = 43/369, 11.7%)
- Adults (18-64 yrs old) (*n* = 308/369, 83.5%)
- Older adults (65+ yrs old) (*n* = 6/369, 1.6%)

9. What is the most common mental health disorder you provide services for?

- Anxiety disorders (*n* = 166/369, 45.0%)
- Mood disorders (*n* = 81/369, 22.0%)
- Trauma- and stressor-related disorders (*n* = 88/369, 23.8%)
- Substance-related and addictive disorders (*n* = 7/369, 1.9%)
- Personality disorders (*n* = 3/369, 0.8%)
- Disruptive, impulse control, and conduct disorders (*n* = 8/369, 2.2%)
- Somatic symptom and related disorders (*n* = 0/369, 0.0%)
- Other (Please specify): ________________ (*n* = 16/369, 4.3%)

10. When did you begin using telemedicine in your practice?

- December 2019 or earlier (*n* = 73/369, 19.8%)
- January or February 2020 (*n* = 39/369, 10.6%)
- March 2020 or later (*n* = 257/369, 69.6%)

11. How are you primarily reimbursed for your telemedicine services?

- Public Insurance (Medicare, Medicaid) (*n* = 63/369, 17.1%)
- Private Insurance (*n* = 243/369, 65.9%)
- Client out-of-pocket (*n* = 63/369, 17.1%)

12. What percent of your clients do you see via telemedicine?

- < 25% (*n* = 31/369, 8.4%)
- 25%–49% (*n* = 39/369, 10.6%)
- 50–75% (*n* = 34/369, 9.2%)
- > 75% (*n* = 265/369, 71.8%)

13. How have your overhead costs (e.g., rent, office supplies, travel) changed as a result of providing telehealth services?

- Greatly decreased (*n* = 47/369, 12.7%)
- Decreased some (*n* = 97/369, 26.3%)
- Haven’t changed (*n* = 169/369, 45.8%)
- Increased some (*n* = 47/369, 12.7%)
- Greatly increased (*n* = 9/369, 2.4%)

|  | 1. Strongly Disagree | 2. Somewhat Disagree | 3. Neither agree nor disagree | 4. Somewhat Agree | 5. Strongly Agree |
| --- | --- | --- | --- | --- | --- |
| 14. I think I would like to use telemedicine frequently in my practice | 11 (3.0%) | 41 (11.1%) | 29 (7.9%) | 119 (32.2%) | 169 (45.8%) |
| 15. I find telemedicine unnecessarily complex | 204 (55.3%) | 107 (29.0%) | 34 (9.2%) | 17 (4.6%) | 7 (1.9%) |
| 16. I think telemedicine is easy to use in my practice | 8 (2.2%) | 18 (4.9%) | 22 (6.0%) | 152 (41.2%) | 169 (45.8%) |
| 17. I think I need more technical support to effectively use telemedicine | 141 (38.2%) | 99 (26.8%) | 58 (15.7%) | 55 (14.9%) | 16 (4.3%) |
| 18. I find the various functions in the telemedicine platform are well integrated | 4 (1.1%) | 38 (10.3%) | 101 (27.4%) | 145 (39.3%) | 81 (22.0%) |
| 19. I think there is too much inconsistency in the telemedicine platform | 84 (22.8%) | 82 (22.2%) | 100 (27.1%) | 89 (24.1%) | 14 (3.8%) |
| 20. I imagine most people learn to use telemedicine very quickly | 3 (.8%) | 34 (9.2%) | 65 (17.6%) | 177 (48.0%) | 90 (24.4%) |
| 21. I find telemedicine very complicated to use | 231 (62.6%) | 103 (27.9%) | 19 (5.1%) | 13 (3.5%) | 3 (.8%) |
| 22. I feel confident using telemedicine in my practice | 5 (1.4%) | 13 (3.5%) | 21 (5.7%) | 125 (33.9%) | 205 (55.6%) |
| 23. I needed to learn a lot of things before I could get going with telemedicine | 160 (43.4%) | 97 (26.3%) | 42 (11.4%) | 54 (14.6%) | 16 (4.3%) |

How beneficial do you believe that telemedicine services have been to…

|  | 1. Not at all | 2. Slightly | 3. Moderately | 4. Very | 5. Extremely |
| --- | --- | --- | --- | --- | --- |
| 24. Limiting the spread of COVID-19 | 3 (.8%) | 12 (3.3%) | 26 (7.0%) | 77 (20.9%) | 251 (68%) |
| 25. Protecting the safety of you and your clients | 2 (.5%) | 9 (2.4%) | 14 (3.8%) | 82 (22.2%) | 262 (71.0%) |
| 26. Increasing clients’ access to care | 2 (.5%) | 11 (3.0%) | 33 (8.9%) | 94 (25.5%) | 229 (62.1%) |
| 27. Reducing client no-show rates | 30 (8.1%) | 32 (8.7%) | 81 (22.0%) | 96 (26.0%) | 130 (35.2%) |
| 28. Reducing costs/overhead of your practice | 137 (37.1%) | 45 (12.2%) | 82 (22.2%) | 46 (12.5%) | 59 (16.0%) |
| 29. Improving your work-life balance | 81 (22.0%) | 73 (19.8%) | 79 (21.4%) | 65 (17.6%) | 71 (19.2%) |

When you think about your experience using telemedicine during COVID-19, do you feel:

|  | 1. Strongly Disagree | 2. Somewhat Disagree | 3. Neutral | 4. Somewhat Agree | 5. Strongly Agree |
| --- | --- | --- | --- | --- | --- |
| 30. Supported to practice your specialty via telemedicine? | 4 (1.1%) | 21 (5.7%) | 33 (8.9%) | 142 (38.5%) | 169 (45.8%) |
| 31. Adequately trained to practice your specialty via telemedicine? | 10 (2.7%) | 31 (8.4%) | 43 (11.7%) | 134 (36.3%) | 151 (40.9%) |
| 32. You have resources to effectively practice your specialty via telemedicine? | 8 (2.2%) | 20 (5.4%) | 39 (10.6%) | 151 (40.9%) | 151 (40.9%) |
| 33. Other providers and support staff (e.g., nurses) are comfortable referring their patients to a provider who uses telemedicine? | 7 (1.9%) | 7 (1.9%) | 95 (25.7%) | 121 (32.8%) | 139 (37.7%) |

The two items below ask about telemedicine use after the COVID-19 pandemic.

|  | 1. Much less | 2. Somewhat less | 3. About the same | 4. Somewhat more | 5. Much more |
| --- | --- | --- | --- | --- | --- |
| 34. After the COVID-19 pandemic is resolved, I expect **telemedicine** to continue to be used by others in my profession: | 28 (7.6%) | 87 (23.6%) | 61 (16.5%) | 70 (19.0%) | 123 (33.3%) |
| 35. After the COVID-19 pandemic is resolved, I expect to use **telemedicine in my practice**: | 54 (14.6%) | 76 (20.6%) | 66 (17.9%) | 75 (20.3%) | 98 (26.6%) |
