# Supplementary material for: Providers’ Perspectives on Telemental Health Usage After the COVID-19 Pandemic: Retrospective Analysis
Source: JMIR Form Res. 2022 Nov 11;6(11):e39634. doi: 10.2196/39634 (PMC9662289; doi:10.2196/39634)
Supplement: Multimedia Appendix 2 [file formative_v6i11e39634_app2.docx]

**Multimedia Appendix 2.**

Correlation table (Pearson *r* and two-tailed *P* value) of practice characteristics and measures.

| Variable | 1. | 2. | 3. | 4. | 5. | 6. | 7. | 8. | 9. |
| --- | --- | --- | --- | --- | --- | --- | --- | --- | --- |
| **1. Intentions to Continue Using Telemedicine Post-COVID** | | | | | | | | | |
| *r* | 1 | .21** | -.13* | -.11* | .39** | .28** | .31** | .30** | .77** |
| *P* value | - | < .001 | < .001 | < .001 | < .001 | < .001 | < .001 | < .001 | < .001 |
| **2. Telemedicine Caseload** | | | | | | | | | |
| *r* | .21** | 1 | .06 | -.06 | .24** | .26** | .12* | .06 | .10* |
| *P* value | < .001 | - | .20 | .20 | < .001 | < .001 | .01 | .21 | .05 |
| **3. Onset of telemedicine usage** | | | | | | | | | |
| *r* | -.13* | .06 | 1 | .03 | -.05 | .01 | -.09* | -.12* | -.08 |
| *P* value | .01 | .20 | - | .51 | .33 | .89 | .05 | .01 | .10 |
| **4. Age** | | | | | | | | | |
| *r* | -.11* | -.06 | .03 | 1 | -.05 | -.16** | -.07 | -.04 | -.12* |
| *P* value | .03 | .20 | .51 | - | .35 | < .001 | .13 | .37 | .02 |
| **5. Perceived Usefulness (General)** | | | | | | | | | |
| *r* | .39** | .24** | -.05 | -.05 | 1 | .44** | .32** | .32** | .31** |
| *P* value | < .001 | < .001 | .33 | .35 | - | < .001 | < .001 | < .001 | < .001 |
| **6. Perceived Usefulness (COVID-19)** | | | | | | | | | |
| *r* | .28** | .26** | .01 | -.16** | .44** | 1 | .24** | .31** | .22** |
| *P* value | < .001 | < .001 | .89 | < .001 | < .001 | - | < .001 | < .001 | < .001 |
| **7. Perceived Ease-of-Use** | | | | | | | | | |
| *r* | .31** | .12* | -.09* | -.07 | .32** | .24** | 1 | .49** | .21** |
| *P* value | < .001 | .01 | .05 | .13 | < .001 | < .001 | - | < .001 | < .001 |
| **8. Facilitating Conditions** | | | | | | | | | |
| *r* | .30** | .06 | -.12* | -.04 | .32** | .31** | .49** | 1 | .20^**^ |
| *P* value | < .001 | .21 | .01 | 0.37 | < .001 | < .001 | < .001 | - | < .001 |
| **9. Social Influence** | | | | | | | | | |
| *r* | .77** | .10* | -.08 | -.12* | .31** | .22** | .21** | .20** | 1 |
| *P* value | < .001 | .05 | .10 | .02 | < .001 | < .001 | < .001 | < .001 | - |

*Note.* * indicates *p* < .05; ** indicates *p* < .01.
